# Association between handgrip strength and bone mass parameters in HIV-infected children and adolescents. A cross-sectional study

**DOI:** 10.1590/1516-3180.2020.0539.R1.090321

**Published:** 2021-06-25

**Authors:** Priscila Custódio Martins, Luiz Rodrigo Augustemak de Lima, Tiago Rodrigues de Lima, Edio Luiz Petroski, Diego Augusto Santos Silva

**Affiliations:** I MSc. Doctoral Student, Department of Physical Education, Universidade Federal de Santa Catarina (UFSC), Florianópolis (SC), Brazil.; II PhD. Adjunct Professor, Instituto de Educação Física e Esporte (IEFE), Universidade Federal de Alagoas (UFAL), Maceió (AL), Brazil.; III MSc. Doctoral Student, Department of Physical Education, Universidade Federal de Santa Catarina (UFSC), Florianópolis (SC), Brazil.; IV MSc, PhD. Full Professor, Department of Physical Education, Universidade Federal de Santa Catarina (UFSC), Florianópolis (SC), Brazil.; V MSc, PhD. Associate Professor, Department of Physical Education, Universidade Federal de Santa Catarina (UFSC), Florianópolis (SC), Brazil.

**Keywords:** Body composition, Bone and bones, Child health, Adolescent health, Body fat, Children’s health, Teen health

## Abstract

**BACKGROUND:**

Low bone mineral content (BMC) and bone mineral density (BMD) have been identified in human immunodeficiency virus (HIV)-infected children and adolescents. The direct adverse effects of HIV infection and combined antiretroviral therapy (ART) negatively contribute to bone metabolism. A direct relationship between muscle strength levels and BMD in HIV-infected adults and older adults has been described. However, it is unknown whether handgrip strength (HGS) is associated with bone mass in pediatric populations diagnosed with HIV.

**OBJECTIVE:**

To ascertain whether HGS levels are associated with BMC and BMD in HIV-infected children and adolescents.

**DESIGN AND SETTING:**

Cross-sectional study conducted in Florianãpolis, Brazil, in 2016.

**METHODS:**

The subjects were 65 children and adolescents (8-15 years) diagnosed with vertically-transmitted HIV. Subtotal and lumbar-spine BMC and BMD were obtained via dual-emission X-ray absorptiometry (DXA). HGS was measured using manual dynamometers. The covariates of sex, ART, CD4+ T lymphocytes and viral load were obtained through questionnaires and medical records. Sexual maturation was self-reported and physical activity was measured using accelerometers. Simple and multiple linear regression were used, with P < 0.05.

**RESULTS:**

HGS was directly associated with subtotal BMD (β = 0.002; R² = 0.670; P < 0.001), subtotal BMC (β = 0.090; R² = 0.734; P = 0.005) and lumbar-spine BMC (β = 1.004; R² = 0.656; P = 0.010) in the adjusted analyses. However, no significant association was found between HGS and lumbar-spine BMD (β = 0.001; R² = 0.464; P = 0.299).

**CONCLUSION:**

HGS was directly associated with BMD and BMC in HIV-infected children and adolescents.

## INTRODUCTION

Low bone mineral content (BMC) and bone mineral density (BMD) have been identified in human immunodeficiency virus (HIV)-infected children and adolescents.^1^ This occurs because of the direct adverse effects of HIV infection and combined antiretroviral therapy (ART), which negatively contribute to bone metabolism.^2,3,4^ The marked reductions in BMC and BMD are directly associated with increased proinflammatory cytokines, delayed growth and maturational development, decreased muscle mass levels and endocrine disorders, which reduce osteoblast activity and stimulate osteoclasts.^2,3^

Damage to bone mass is especially worrisome in pediatric populations, whether diagnosed with HIV or not, since during this phase of life, there is greater bone mineral increase.^5^ Approximately 85%-90% of final adult bone mass is reached during childhood and adolescence, and losses from bone accumulation during this critical period may compromise peak bone mass, which is recognized as the determinant for the risks of osteoporosis and fractures in adulthood.^6^

Although major efforts to attenuate bone loss have been based on pharmacological interventions (bisphosphonates)^7^ and vitamin D and calcium supplementation, non-pharmacological strategies based on physical activity that promote gravitational overload (running and jumping) or muscle tension (strength training) have the capacity to induce bone formation stimuli and inhibit bone resorption.^8,9^ Handgrip strength (HGS) enhancement exercises have been suggested as a strategy in HIV treatment because they improve cardiorespiratory fitness, body composition and weight control.^10^ In the literature, a direct relationship between HGS levels and BMD in HIV-infected adults^11,12^ and older adults^12^ has already been described. However, it is unknown whether HGS is associated with bone mass in pediatric populations diagnosed with HIV.

Investigations relating to the association between HGS and bone mass in HIV-infected children and adolescents are important and can support non-pharmacological interventions to reduce complications resulting from infection and prolonged use of ART, with regard to functional capacity and body composition (bone mass, fat mass, etc.). These complications may include development of osteopenia, early osteoporosis and, possibly, related fractures due to falls, which would increase the risk of morbidity in adulthood. In children and adolescents without a diagnosis of HIV, HGS has been described as a marker of bone health and overall health.^8^ Among the strategies available for measuring HGS levels, use of manual dynamometers has been highlighted as a simple and widely applicable method, due to its low cost, speed and association with general HGS.^13^

## OBJECTIVE

The aim of the present study was to ascertain whether HGS levels are associated with BMC and BMD in HIV-infected children and adolescents.

## METHODS

### Study design

The present study was conducted in the city of Florianãpolis (SC), Brazil, in 2016. The study was approved by the Research Ethics Committee of the Universidade Federal de Santa Catarina (UFSC) (protocol number 49691815.0.0000.0121; date: October 10, 2015). All parents/guardians of the children and adolescents signed an informed consent statement authorizing participation in the research. 

### Participants

Children and adolescents (8 to 15 years old) with a diagnosis of HIV that had been acquired via vertical transmission, who were under clinical follow-up at the “Joana de Gusmã” Children’s Hospital, were recruited for the study. The eligibility criteria were the following: a) presentation of a record of HIV infection by means of vertical transmission in the medical records; b) age between 8 and 15 years; c) availability of clinical and laboratory information in the medical records; and d) ability to stand and to communicate. The exclusion criteria were the following: a) motor impairment or contraindication for vigorous exercise; b) speech, hearing and/or cognition impairment; c) presence of diseases that alter body composition, except for HIV infection; and d) occurrence of regular use of diuretic medications or immunotherapies. Participants presenting any pathological condition other than HIV infection that changed body composition were excluded from the study.

### Dependent variables

Total, subtotal (all sites except head) and lumbar-spine BMD and BMC were obtained by means of dual emission X-ray absorptiometry (DXA) (Lunar Prodigy Advance Model Discovery Wi-Fan-Beam - S/N 81593; GE Medical Systems, Madison, United States). X-ray attenuation in body tissues was computed using the Encore 13.60.033 software, pediatric version 8.10.027 (GE Medical Systems, Madison, United States). Internal quality control was ensured by performing a standard daily calibration process provided by the manufacturer of the DXA machine. During the DXA assessments, the participants were barefoot, wearing appropriate clothing and not wearing any metallic accessories.^14^ The reading of the biometric pattern by means of a specific body-wide sensor (head to toe) lasted approximately 10 minutes, while the individual remained supine on the device stretcher with arms extended along the sides of the body, with palms facing downwards.^15,16^

### Independent variable

HGS was measured using a Saehan dynamometer (Model SH5001, Saehan Corporation, Masan, Korea), which has been concurrently validated with the Jamar dynamometer (r = 0.976), with high intra-examiner reliability (r = 0.985).^17^ This test was chosen because HGS levels have been strongly correlated with total muscle strength (correlation coefficient 0.736 to 0.890) in children, adolescents and adults.^18^

The evaluation procedures followed the protocol described by the Canadian Society for Exercise Physiology.^19^ During the evaluation, the participants were instructed to stand with their arms extended at their sides, holding the device in one hand without it touching the corresponding thigh. The device was gripped between the distal phalanges and the palm of the hand.^19^ The subject was asked to breathe in and then exhale as much as possible, followed by exerting greater pressure with the hand on the device. The test was performed twice in each hand, alternating hands between tests. The full strength was calculated as the sum of the largest reading from each hand.

### Control variables

Sex (male or female) was ascertained at interviews. Information on CD4+ T lymphocyte count (%), HIV viral load (logarithmic) and antiretroviral therapy (ART) (categorized as not using ART, using ART without protease inhibitors (PI) or using ART with PI) was obtained from the medical records. These variables relating to treatment and infection were used as controls in analyses because previous studies had identified an inverse association with bone mass.^1^ Sexual maturation was self-reported by the participants by using pubic hair images, in accordance with Tanner’s procedures.^20^

Moderate to vigorous-intensity physical activity (MVPA) was investigated using the Actigraph accelerometer (model GT3X-Plus; Manufacturing Technology Inc., Fort Walton Beach, United States), with continuous use for 7 to 14 days, including weekends. The participants were instructed to wear the equipment on the right side of the waistline from early morning until the end of the day, taking it off only during water activities and sleep. For the data analysis, records of at least four days (three on weekdays and one on weekends) for a period of 10 hours or more on each day, after removal of non-use time of at least 60 consecutive zeros (60 minutes), were taken into consideration. The number of minutes of MVPA minutes was obtained from cutoff points described by Evenson^21^ and was proportionally adjusted to the average length of time for which the adolescents stayed awake (14 hours). Verbal and written instructions were made available to participants and guardians before the device was used. MVPA was used as the adjustment variable because less physically active HIV+ individuals tend to have lower BMD and BMC values.^22^

### Statistical analysis

Descriptive analysis (median and interquartile range) was performed on the data. Kurtosis and asymmetry were used to verify data normality (range from -2 to + 2), and histogram analysis was used to identify normality in data distribution. Pearson’s linear correlation and multiple linear regression were used to test correlations and associations between outcome and exposure, respectively. For multiple regression analysis, control variables (sex, sexual maturation, skin color, viral load, type of medication used and level of habitual physical activity) were entered. Regression coefficients (β), 95% confidence interval and determination coefficients for each model analyzed (R²), multicollinearity diagnosis (VIF), Akaike information criterion (AIC), Bayesian information criterion (BIC) and effect size (Cohen’s D) were estimated. For all analyses, the STATA software, version 14.0 (StataCorp LLC, College Station, Texas, United States), was used. The statistical significance level was set at P ≤ 0.05.

## RESULTS

Sixty-five adolescents (30 males and 35 females) aged 8-15 years who had been diagnosed with HIV participated in the study. The participants’ characteristics are highlighted in **Table 1**.

**Table 1.** Characteristics of the children and adolescents diagnosed with human immunodeficiency virus (HIV)

	Median	Interquartile range (p25; p75)
Chronological age (years)	12.7	10.5; 14.0
Bone age (years)	12.5	10.0; 14.0
Height (cm)	149.8	139.3; 156.4
Body mass (kg)	38.4	31.7; 49.6
Body mass index (kg/m^2^)	17.4	16.0; 19.9
LSTM (kg)	29.0	23.4; 33.8
BMD total (g/cm^2^)	0.930	0.881; 1.007
BMC total (g)	1461.9	1158.9; 1819.0
Z-score BMD total (SDS)	-0.028	-0.242; 0.146
Z-score BMC total (SDS)	-0.332	-0.991; 0.709
BMD subtotal (g/cm^2^)	0.823	0.756; 0.912
BMC subtotal (g)	1108.8	848.3; 1449.3
BMC subtotal/height (g/cm)	7.481	6.106; 9.152
BMD lumbar spine (g/cm^2^)	0.801	0.707; 0.889
BMC lumbar spine (g)	121.5	85.1; 159.6
Handgrip strength (kg)	19.0	14.0; 26.0
Moderate-vigorous physical activity (min/day)	43.8	26.6; 64.2
Viral load (log)	1.6	1.6; 2.5
CD4+ T lymphocytes (cell/mm^3^)	819.0	575.0; 1091.0
CD8+ T lymphocytes (cell/mm^3^)	1088.0	790.0; 1422.0
	**n**	**(%)**
**Skin color**
White	29	44.6
Brown, black, East Asian and indigenous	36	55.4
**Current ART**
Yes, with PI	39	60.0
Yes, without PI	15	23.1
Not used	11	16.9
**Sexual maturation**
Pre-puberty	15	23.1
Puberty	47	72.3
Post-pubertal	03	4.6

n = sample; % = percentage; g = grams; kg = kilograms; cm = centimeters; mm = millimeters; min = minutes; p25 = 25^th^ percentile; p75 = 75^th^ percentile; ART = antiretroviral therapy; PI = protease inhibitors; BMD = bone mineral density; BMC = bone mineral content; LSTM = lean soft tissue mass; SDS = standard deviation score.

**Figure 1.**
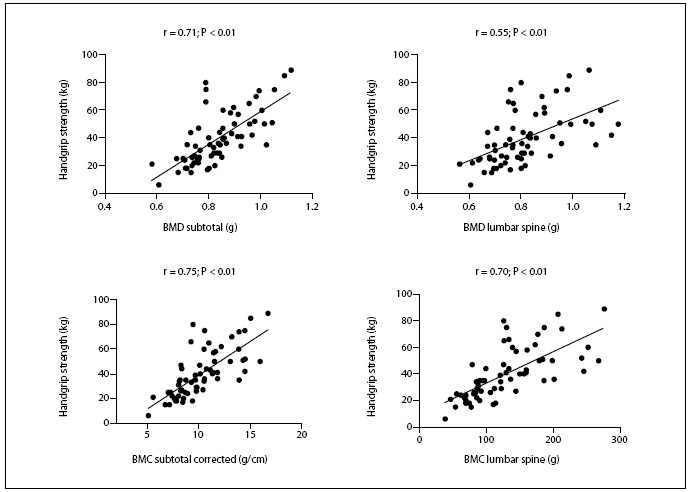
Pearson’s linear correlation for total and regional bone parameters (bone mineral density [BMD] and bone mineral content [BMC]) in relation to handgrip strength, among children and adolescents diagnosed with HIV (n = 65).

Pearson’s linear correlation analysis demonstrated that HGS was directly correlated with total BMD (r = 0.60; P < 0.01), total BMC (r = 0.77; P < 0.01) and subtotal BMC (r = 0.78; P < 0.01). **Figure 1** shows correlations between HGS and subtotal BMD (r = 0.71; P < 0.01) and lumbar-spine BMD (r = 0.55; P < 0.01) in these HIV-infected children and adolescents, along with the association between HGS and subtotal BMC corrected for height (r = 0.75; P < 0.01) and lumbar-spine BMC (r = 0.70; P < 0.01).

Single and multiple linear regression analyses are presented in **Table 2**. In these HIV-infected children and adolescents, HGS was directly associated with subtotal BMD (β = 0.002; P < 0.001), even after adjustment for sex, sexual maturation, skin color, viral load, type of medication used and level of habitual physical activity. HGS was directly associated with lumbar-spine BMD (β = 0.003; P < 0.001), but after adjusting for covariates, the association lost statistical significance. Regarding subtotal BMC and lumbar spine BMC, single and multiple analyses showed direct associations with HGS (β = 0.090 and P = 0.005; and β = 1.004 and P = 0.010, respectively).

**Table 2.** Simple and multiple linear regression on bone mineral density and bone mineral content in relation to handgrip strength in children and adolescents diagnosed with human immunodeficiency virus (HIV)

	β (95% CI)	β standardized	R^2^	P	VIF	AIC*n	BIC	Cohen’s D
**BMD subtotal**								
HGS	0.004 (0.003; 0.005)	0.711	0.500	**< 0.001**				
Adjusted model	0.002 (0.001; 0.003)	0.380	0.670	**0.003**	1.75	-156.619	-398.348	2.03
**BMD lumbar spine**								
HGS	0.003 (0.002; 0.005)	0.550	0.290	**< 0.001**				
Adjusted model	0.001 (-0.001; 0.002)	0.166	0.464	0.299	1.75	-103.253	-344.982	0.85
**BMC subtotal** ^*^								
HGS	0.096 (0.075; 0.118)	0.755	0.563	**< 0.001**				
Adjusted model	0.053 (0.025; 0.080)	0.427	0.734	**0.001**	1.75	213.388	-28.341	2.70
**BMC lumbar spine**	
HGS	2.086 (1.554; 2.618)	0.702	0.485	**< 0.001**				
Adjusted model	1.004 (0.246; 1.762)	0.351	0.656	**0.010**	1.84	629.718	387.989	1.86

HGS = handgrip strength; CI = confidence interval; VIF = diagnosis of multicollinearity; AIC*n = Akaike information criterion; BIC = Bayesian Information Criterion; BMD = bone mineral density; BMC = bone mineral content; BMC subtotal* = corrected for height; Adjusted model = adjusted for sex, sexual maturation, skin color, viral charge, type of medicine used, CD4+ T lymphocytes and level of habitual physical activity.

## DISCUSSION

The main findings from the present study were that HGS was directly associated with BMD and BMC (total, subtotal and lumbar-spine) in HIV-infected children and adolescents. These results are unprecedented in the pediatric population diagnosed with HIV and demonstrate the importance of HGS for bone mass. In HIV-infected adults, it has been shown in the literature that HGS was directly associated with different health outcomes, such as cardiometabolic risk,^12^ phase angle^23^ and overall quality of life.^24^

Our results were consistent with those from previous studies among adults^11,12^ and older adults^11^ diagnosed with HIV. In those studies, a direct relationship was found between greater HGS and better indicators for bone parameters. The present study showed that this relationship is also true for HIV-infected children and adolescents.

In the present study, the physiological mechanisms that explain the direct association between HGS levels and bone mass were not evaluated, but the results can be explained in terms of several factors. First, the mechanostatic theory postulates that HGS is a predictor of bone mass in different populations.^25,26^ With the exception of trauma, muscles cause the largest loads and deformations on major bones, with potential osteogenic stimulation, which helps control the biological mechanisms that determine bone strength. This promotes bone structure strength in children and adolescents, depending on increasing HGS and how bones respond to it.^27^ Second, hormones and other non-mechanical agents that affect bone strength may affect the muscle-bone unit strength relationship; however, the effect that HGS exerts on bone strength cannot be replaced. In addition, some biological agents that directly exert effects on bones through actions on bone cells (e.g. growth hormones, androgenic hormones, calcium, vitamin D and their metabolites) also perform different functions that stimulate HGS.^27^ Third, activities that develop HGS positively contribute to leptin secretion, which is directly related to greater stimulation, proliferation and differentiation of osteoblasts and osteoclasts. These provide the best balance in the process of absorption and remodeling of bone structures.^28^

However, even though HGS and bone mass are directly interrelated, thus suggesting the possibility of interventions consisting of physical exercise, children and adolescents with chronic conditions may have restrictions on participating in more intense physical activity, due to both real and perceived limitations.^29^ In fact, in previous research using the same sample as in the present study, Lima et al.^30^ identified that HIV-infected children and adolescents accumulated lower bouts of moderate-to-vigorous physical activity, compared with healthy controls. This may result in reduced total physical activity, which in turn may impair the development of HGS and skeletal impact load, thereby compromising full bone development.^3,4^ In addition, ART may negatively affect bone parameters, which may compromise bone health.^2^ However, correct and continuous use of ART is indispensable, as it enables longer survival for HIV patients. Although there is no consensus regarding the volume and intensity of muscle strength exercises and habitual physical activity that are necessary for mitigating the negative effects of ART,^7^ strategies aimed at improving muscle strength, as well as increasing the time spent on physical activity, are necessary.

There is a consensus that physical activity is important for bone mass in the first two decades of life because it assists in the mineralization process of the growing bones.^22^ Moreover, structural improvements can be achieved by increasing mechanical load through habitual physical activity.^22^ Involvement in activities that require maximum strength or resistance (for example, weight lifting) or those involving large body motion (for example, individual or team sports with high-intensity motion) can lead to increased stimulation of the bone matrix. Thus, it might be theorized that physical activities, especially those requiring HGS, can attenuate or even reverse the deleterious effect of HIV and ART on bone mass, which would minimize the subsequent risk of osteoporosis in adulthood.^27^ However, exercise-based intervention studies that simulate strength are needed to test this hypothesis.

In the present study, HGS presented lower explanatory power regarding lumbar-spine BMD variability, compared with whole-body BMD. Trabecular bone (e.g. the lumbar spine) is mainly affected by general and systemic factors such as hormonal status. However, cortical bone (e.g. femurs) is more likely to be affected by regional mechanical influences, such as the force of gravity, muscle mass and strength, which may explain the lower explanatory power of HGS regarding lumbar-spine BMD, compared with whole-body BMD.

This research had limitations that suggest that caution is needed in interpreting the results, such as its sample heterogeneity due to the variability of age, clinical condition and use of ART. The lack of information about calcium and vitamin D intake and blood concentration may also be considered to be a study limitation. In addition, the cross-sectional design does not allow cause-and-effect inferences among study variables. The study also had strengths such as the precision and reliability of the instruments used, in addition to the representativeness of the pediatric patients in the region researched (78% of the patients were treated at the reference hospital).

## CONCLUSIONS

It was concluded that HGS was directly associated with BMD and BMC in HIV-infected children and adolescents, regardless of sex, sexual maturation, skin color, viral load, type of medication used and level of habitual physical activity. Randomized clinical trials should be carried out to confirm whether exercises that stimulate HGS promote bone mass improvements in HIV-infected children and adolescents.

### Practical applications

The results from the present study demonstrated the direct association between HGS levels and bone mass in HIV-infected children and adolescents. Based on these results, physical activity promotion strategies aimed at improving HGS levels can be designed to contribute to maintenance or improvement of bone mass parameters. Physical activities that increase strength levels, such as those performed with one’s body mass (calisthenics), performed individually (e.g. rope jumps, push-ups or bar pulls) or in pairs/groups (e.g. wheelbarrow, tug of war or arm wrestling) can be encouraged by healthcare and exercise professionals working with HIV-infected children and adolescents. These can form an integral part of non-pharmacological strategies aimed at maintaining or improving bone parameters in this population.
